# Serum for Tetanus

**Published:** 1896-05

**Authors:** 


THE *Veterinarian* reports the success of a French veterinary surgeon in obtaining recovery in two cases of tetanus with serum taken from an aged horse suffering from severe tetanus. 5 and 10 per cent. solutions, with distilled water, were used; 120 grammes, in six injections, were given—in one case in the thigh, in the second case in the gluteal region.

